# Supplementary material for: High moon brightness and low ambient temperatures affect sloth predation by harpy eagles
Source: PeerJ. 2020 Aug 27;8:e9756. doi: 10.7717/peerj.9756 (PMC7456529; doi:10.7717/peerj.9756)
Supplement: Supplemental Information 2 — Each GLMM is used to predict the probability that a given prey item belongs to the response variable. ‘Normalized difference vegetation index’ (NDVI) is a proxy for deciduousness, calculated using data from LANDSAT imagery. All other climatic variables were obtained from meteorological stations near the study site. Variables noted as ‘three-day’ are pooled over a three-day period up to any given prey detection event. [file peerj-08-9756-s002.docx]

**Table S1. Variables used in the models.**

Climatic variables used in mixed generalized linear models (GLMM) explaining the dietary profile of harpy eagles. Each GLMM is used to predict the probability that a given prey item belongs to the response variable. ‘Normalized difference vegetation index’ (NDVI) is a proxy for deciduousness, calculated using data from LANDSAT imagery. All other climatic variables were obtained from meteorological stations near the study site. Variables noted as ‘3-day’ are pooled over a three-day period up to any given prey detection event.

| **Explanatory Variables** | **Response Variable** | | |
| --- | --- | --- | --- |
|  | **Sloth** | **Nocturnal** |  |
| NDVI | X | X |  |
| Cumulative rainfall (3-Day; mm) | X |  |  |
| Rainfall (mm) | X |  |  |
| Minimum temperature (3-Day; °C) | X |  |  |
| Minimum temperature (°C) | X |  |  |
| Maximum temperature (°C) | X |  |  |
| Lunar disc (%) | X | X |  |
| Maximum lunar disc (3-Day; %) |  | X |  |
| Minimum lunar disc (3-Day; %) |  | X |  |
| Deciduousness*Maximum lunar disc | X | X |  |
| Cumulative rainfall*Minimum temperature (3-day) | X |  |  |
