# Supplementary figures and images for: High moon brightness and low ambient temperatures affect sloth predation by harpy eagles
Source: PeerJ. 2020 Aug 27;8:e9756. doi: 10.7717/peerj.9756 (PMC7456529; doi:10.7717/peerj.9756)

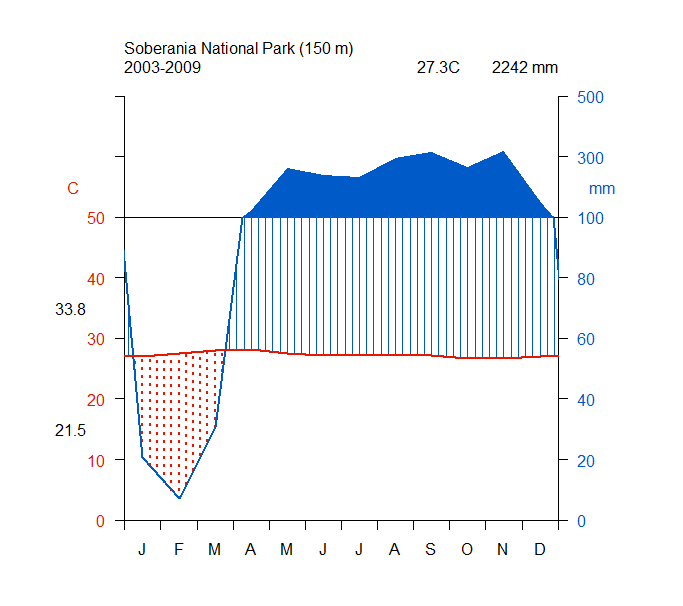

Supplement: Supplemental Information 3 — Walter-Lieth diagram showing precipitation and temperatures at the study site. [file peerj-08-9756-s003.png]

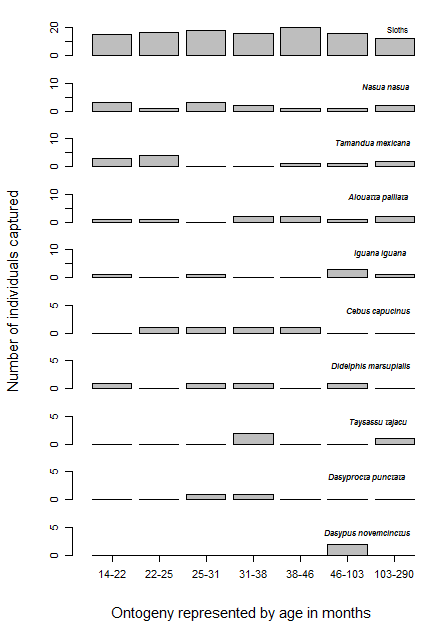

Supplement: Supplemental Information 4 — Number and species of individuals captured along ageing in reintroduced harpy eagles. [file peerj-08-9756-s004.png]

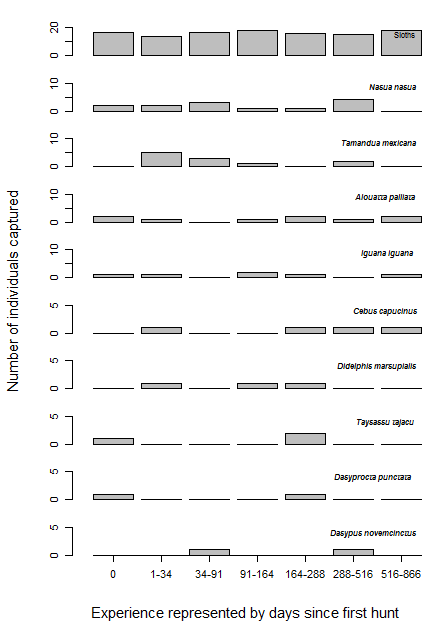

Supplement: Supplemental Information 5 — Harpy eagle experience in days and prey number and species. No effect of greater experience can be seen in prey composition. [file peerj-08-9756-s005.png]
